# Current Melioidosis Diagnostic Landscape and Missed Opportunities in Biomarker Development

**DOI:** 10.3390/diagnostics16081247

**Published:** 2026-04-21

**Authors:** Sri Agung Fitri Kusuma, Santi Rukminita Anggraeni, Qurnia Wulan Sari, Neng Tanty Sofyana

**Affiliations:** 1 Department of Pharmaceutical Biology, Faculty of Pharmacy, Padjadjaran University, Sumedang 45363, West Java, Indonesia; 2 Marine Department, Faculty of Fisheries and Marine Sciences, Padjadjaran University, Sumedang 45363, West Java, Indonesia; santi.rukminita@unpad.ac.id (S.R.A.); qurnia.w.sari@unpad.ac.id (Q.W.S.); neng.tanty@unpad.ac.id (N.T.S.)

**Keywords:** melioidosis, *Burkholderia pseudomallei*, short peptide biomarkers, diagnostic biomarkers, capsular polysaccharide, type III secretion system 1, rapid diagnostics, peptide-based diagnostics

## Abstract

**Background/Objectives**: Melioidosis, caused by *Burkholderia pseudomallei*, is a severe tropical infectious disease associated with high mortality in endemic regions. Early diagnosis remains challenging because conventional diagnostic methods, including culture, serological assays, and molecular techniques, have limitations in sensitivity, specificity, processing time, and accessibility in resource-limited settings. This review evaluates current diagnostic approaches and highlights the potential of short peptide biomarkers for improving melioidosis detection. **Methods**: A narrative literature review was conducted using four electronic databases (PubMed, Scopus, Web of Science, and Google Scholar) covering publications from 2000 to 2024. Relevant studies were identified using predefined keywords related to melioidosis diagnostics, biomarkers, and peptide-based approaches, and were screened based on relevance to diagnostic methods and peptide biomarker development in *Burkholderia pseudomallei*. **Results**: Several biomarkers have been investigated for melioidosis diagnostics, including capsular polysaccharide (CPS), type III secretion system 1 (TTS1), and other virulence-associated proteins such as Hcp1 and BPSS1187. Among these, CPS and TTS1 are highly conserved and specific targets widely used in molecular and antigen-based detection methods. Short peptide epitopes derived from these antigens demonstrate promising advantages over whole proteins, including improved stability, high specificity, easier synthesis, and reduced production costs. Advances in epitope prediction technologies and peptide-based biosensors have further expanded the potential applications of short peptides in rapid diagnostic platforms, including ELISA, lateral flow immunoassays, and biosensor-based detection systems. **Conclusions**: Short peptide–based biomarkers represent a promising strategy for developing rapid, sensitive, and cost-effective diagnostic tools for melioidosis, particularly in endemic and resource-limited settings.

## 1. Introduction

Melioidosis is a neglected tropical disease caused by *Burkholderia pseudomallei* and imposes a considerable global health burden. The disease is endemic in more than 45 countries, particularly in Southeast Asia and northern Australia, with recent reports confirming its emergence in previously non-endemic regions, including the southern United States, Africa, and the Pacific [1,2]. In endemic areas, *B. pseudomallei* infections frequently result in septicemia and pneumonia, leading to mortality rates ranging from 19% to 36%, and globally between 9% and 70%. Fatality rates can increase to 40–50% when patients do not receive prompt and appropriate treatment [3,4,5]. Despite its significant public health impact, melioidosis remains excluded from the World Health Organization (WHO) list of neglected tropical diseases [6]. This omission highlights the global under-recognition of the disease, compounded by the lack of reliable incidence and prevalence data in the WHO Global Health Estimates [7]. Consequently, this knowledge gap limits the development of effective prevention and control strategies. Nevertheless, WHO’s support for the 8th World Melioidosis Congress in 2016 reflects increasing awareness of the disease.

The underdiagnosis of melioidosis is further exacerbated by the limited availability of diagnostic infrastructure in endemic regions. Because its clinical manifestations are nonspecific and often resemble other infections such as tuberculosis, delays in diagnosis are common and frequently fatal [8,9,10]. Conventional diagnostic approaches, including microbiological culture and serological assays, remain the primary methods but have well-documented limitations in speed, sensitivity, and specificity. Although culture is considered the gold standard, it is time-consuming (3–7 days) and often impractical for urgent clinical decision-making [11,12]. Rapid diagnostic methods such as lateral flow immunoassays (LFIA) have been introduced to address these limitations; however, their performance is often compromised by low sensitivity in non-blood samples and cross-reactivity with non-pathogenic species such as *Burkholderia thailandensis* [13,14].

Accurate and rapid detection, therefore, requires highly specific and reliable biomarkers. Among the currently investigated targets, capsular polysaccharide (CPS) and type III secretion system 1 (TTS1) are the most extensively studied and have demonstrated strong diagnostic relevance in antigen-based and molecular assays, respectively [15,16,17]. CPS is a carbohydrate antigen involved in immune evasion, while TTS1 is associated with host interaction mechanisms [18,19]. Another candidate, BPSS1187, has also been explored as a species-specific molecular target, although its biological function remains incompletely characterized [20]. While these biomarkers are associated with virulence and are conserved across *B. pseudomallei* strains, their diagnostic performance may vary by clinical specimen type and infection stage, underscoring the need for careful evaluation and complementary approaches.

In addition to these established targets, other biomolecules—including hemolysin co-regulated protein 1 (Hcp1), outer membrane protein A (OmpA), lipase A (LipA), and BimA—have been investigated for their potential diagnostic relevance. However, for several of these candidates, available evidence is still limited to immunogenicity or mechanistic studies, and their diagnostic performance has not been consistently validated in clinical settings. This variability underscores the importance of distinguishing between well-established biomarkers and those that remain exploratory.

In recent years, short peptide epitopes derived from these antigens have emerged as promising alternatives for diagnostic development. Compared with full-length proteins, short peptides offer several advantages, including improved stability, high specificity, and cost-effective synthesis. Advances in epitope prediction technologies have further expanded the potential of peptide-based approaches. Therefore, this review aims to evaluate the current landscape of melioidosis diagnostics, with a particular focus on peptide-based biomarkers derived from key antigens such as CPS and TTS1, and to critically assess the level of evidence for other candidate targets. We also discuss epitope prediction strategies and their potential applications in developing rapid, accessible diagnostic tools, particularly in resource-limited settings.

## 2. Methods

This study was conducted as a narrative literature review to evaluate current diagnostic approaches for *B. pseudomallei* and to explore the potential of short peptide biomarkers. A comprehensive literature search was performed using four electronic databases: PubMed, Scopus, Web of Science, and Google Scholar. The search covered publications from 2000 to 2024. Representative search terms included combinations of keywords such as “melioidosis diagnostics”, “*Burkholderia pseudomallei* biomarkers”, “capsular polysaccharide”, “type III secretion system”, “BPSS1187”, and “short peptide epitopes”.

The initial search yielded approximately 130 articles. Titles and abstracts were screened to identify relevant studies, followed by full-text evaluation. Articles were included if they focused on diagnostic methods, biomarker applications, or peptide-based diagnostic strategies related to *B. pseudomallei*. Studies were excluded if they were not peer-reviewed, lacked relevance to diagnostic applications, or focused on unrelated bacterial species.

The selected studies were then categorized into major diagnostic approaches, including culture-based methods, serological assays, molecular techniques, and emerging peptide-based diagnostics. As this review is narrative in nature, no formal systematic review framework or meta-analysis was applied.

## 3. Overview of Diagnostic Methods for Melioidosis

Accurate and timely diagnosis of *B. pseudomallei* infection is essential for effective clinical management and reducing mortality associated with melioidosis. A variety of diagnostic methods are currently used to detect *B. pseudomallei*, each presenting specific strengths and limitations depending on the clinical context and the availability of laboratory resources. Table 1 summarizes the main diagnostic techniques used for detecting *B. pseudomallei*, including their mechanisms, advantages, limitations, sensitivity, and specificity.

Despite the availability of multiple diagnostic approaches, their performance varies depending on sample type, bacterial load, and laboratory infrastructure. Culture remains the gold standard but may exhibit reduced sensitivity in cases with low bacterial burden or poor sample quality [8,17,19,20]. Molecular methods provide high specificity but may be influenced by inhibitors and sample variability [12,17,24,29]. Similarly, rapid diagnostic tests show variable performance across specimen types, particularly in non-bacteremic infections [13,26,27,28]. Therefore, integrated diagnostic strategies combining multiple approaches are often required [12,13,14,17].

### 3.1. Culture Method

Isolation of *B. pseudomallei* from clinical specimens such as blood, sputum, and pus remains the gold-standard method for diagnosing melioidosis due to its high specificity, which reaches ~100% [8,17]. However, its diagnostic performance is highly dependent on sample type and bacterial load. Sensitivity can vary considerably depending on specimen type [32], with reported values as low as 36.1% in throat swab samples [17] and remaining variable across other specimen types. Despite its role as a definitive diagnostic method, culture has several important limitations. The procedure is time-consuming, typically requiring 3–7 days [11,12], which limits its applicability in acute clinical settings. In addition, culture sensitivity may be reduced in cases with low bacterial burden, poor sample quality, or prior antibiotic exposure, increasing the risk of false-negative results [8,17].

Selective media play an important role in improving the recovery of *B. pseudomallei*. Ashdown agar is widely used in endemic regions due to its selective properties; however, its use may introduce significant diagnostic limitations. Notably, gentamicin—commonly included in Ashdown agar as a selective agent—may inhibit the growth of certain *B. pseudomallei* strains. A study from Sarawak reported that up to 86% of clinical isolates were susceptible to gentamicin, resulting in potential failure of growth on gentamicin-containing media and subsequent underdiagnosis [33].

In addition to these limitations, the diagnostic performance of selective media is also influenced by epidemiological context. A study conducted in a low-prevalence setting demonstrated that selective media could improve diagnostic yield by detecting additional culture-positive cases missed with routine media; however, this improvement was relatively modest and was associated with additional costs and resource requirements [34].

These findings indicate that while selective media such as Ashdown agar may enhance detection in certain settings, their effectiveness is not universal and must be adapted to local epidemiological conditions and laboratory capacity. Combined with evidence that gentamicin-sensitive strains can lead to false-negative results [34], these limitations highlight that culture-based diagnosis is highly context-dependent and may fail under specific regional or methodological conditions [33,34].

Another major challenge associated with culture-based diagnosis is the potential for misidentification, particularly in resource-limited settings. Phenotypic similarities between *B. pseudomallei* and closely related species, such as *B*. *thailandensis*, can lead to diagnostic errors. To address this, culture results are often complemented by biochemical or automated identification systems, including API 20NE and VITEK. However, these systems show variable diagnostic accuracy, with sensitivity ranging from 37 to 99% and specificity of ~92% [24,25]. In addition to these challenges, the reliability of culture-based identification is further influenced by the limitations of presumptive and biochemical diagnostic methods. A comparative study demonstrated that commonly used bench identification approaches—such as oxidase testing, gentamicin resistance, and susceptibility profiling—achieved a sensitivity of 97% but a relatively low specificity of 69%, primarily due to misidentification with closely related species such as *B. thailandensis* [35].

Furthermore, automated systems such as API 20NE have shown highly variable performance, with sensitivity reported as low as 37% in some evaluations. These findings highlight that culture-based diagnosis does not solely depend on bacterial isolation but also critically relies on accurate downstream identification methods. In this context, molecular approaches such as PCR have demonstrated superior diagnostic accuracy, with reported sensitivity and specificity approaching 100%, underscoring the limitations of conventional culture-based identification alone [35,36].

Additional evidence further confirms that misidentification remains a critical limitation in culture-based diagnostics, particularly when relying on automated identification systems. A clinical case study demonstrated that *B. pseudomallei* was initially misidentified as *B. cepacia* with 91% probability by the VITEK 2 system, necessitating molecular confirmation for accurate diagnosis [36]. Comparative evaluations of identification systems further reveal substantial variability in performance. The accuracy of VITEK 2 has been reported to range from 19% to 63–81%, whereas the BD Phoenix system misidentified the majority of isolates (34 of 47). Similarly, the API 20NE system demonstrated highly variable accuracy, ranging from 37% to 99%, depending on study conditions. In contrast, alternative approaches such as latex agglutination have demonstrated markedly higher performance, with reported sensitivity of 99.5% and specificity of 100%, while molecular methods such as PCR consistently achieve near-perfect diagnostic accuracy.

Further comparison of commercially available identification systems shows that their performance may be substantially lower when tested against geographically and temporally diverse strain collections. In a preliminary evaluation, API 20NE correctly identified only 35 of 58 *B. pseudomallei* isolates (60%), with 31% misidentified and 9% classified as not identifiable [37]. The RapID NF Plus system performed even more poorly, with 0% correct identification of *B. pseudomallei* isolates; 52% were misidentified, and 48% were not identifiable [37]. These findings are important because they demonstrate that the diagnostic limitations of culture-based methods do not end with bacterial isolation. Even after successful culture recovery, downstream identification may remain unreliable if the diagnostic database does not adequately represent strain diversity. This also explains why the reported performance of systems such as API 20NE varies markedly across studies and settings, and why suspicious isolates still require confirmatory testing in laboratories with limited identification capacity

Taken together, these findings indicate that although culture remains the reference standard, it cannot be considered uniformly definitive in routine clinical practice without reliable confirmatory identification. Errors in post-culture identification may lead to delayed diagnosis or misclassification of *B. pseudomallei*, particularly in non-endemic or resource-limited settings [33,34,35,36,37].

### 3.2. Antigen-Based Serological Method

Serological methods that detect antibodies or antigens remain widely used due to their operational simplicity and rapid turnaround time, particularly in settings with limited laboratory infrastructure [8]. These assays are typically performed on serum samples and are applicable in both endemic and non-endemic populations. However, their diagnostic performance is highly variable and generally limited. As summarized in Table 1, antibody-based assays such as ELISA and IHA exhibit sensitivity of approximately ~50–60% and specificity of ~80–90%, depending on assay design and population characteristics [13,14].

A key limitation of serological methods is their inability to reliably detect early-stage infection. Antibody production requires time to develop, resulting in reduced sensitivity during the acute phase. This limitation is supported by clinical data showing that the sensitivity of IHA at hospital admission is only 56%, with approximately 44% of patients testing negative at initial presentation [38]. Furthermore, 68% of initially seronegative patients subsequently seroconverted, indicating that antibody responses often develop only after disease progression, thereby limiting the usefulness of serology for early diagnosis. In addition, 92% of seropositive patients remain persistently positive after recovery, making it difficult to distinguish active infection from prior exposure [38].

Beyond delayed seroconversion, additional factors further compromise the sensitivity of serological methods. In a cohort of culture-confirmed melioidosis patients, only 51% were seropositive at presentation, and approximately 26% remained persistently seronegative despite confirmed infection, even with serial testing [39]. Notably, bacteremia was associated with negative serological results, suggesting that rapid disease progression and high bacterial burden may limit detectable antibody responses. These findings indicate that serological failure may arise not only from host immune dynamics but also from limitations in antigen composition, where assay antigens may not adequately represent strain diversity [39].

In addition to biological limitations, serological interpretation is further complicated by epidemiological factors. In endemic regions, individuals may have pre-existing antibodies due to environmental exposure, resulting in high baseline seropositivity. Population-based data indicate that seroprevalence can reach approximately 17% in individuals without confirmed clinical disease, highlighting that seropositivity does not necessarily reflect active infection [40]. Moreover, serological diagnosis often requires paired serum samples to confirm seroconversion, as single-time-point testing lacks reliability and may lead to misinterpretation [41].

The limited diagnostic value of serological methods is further supported by comparative studies. In an endemic setting, IHA demonstrated a sensitivity of 73% and a specificity of 64%, whereas rapid immunochromatographic tests (ICT) showed a specificity as low as 47%, indicating a high rate of false-positive results [42]. Even clinical diagnostic criteria exhibited low specificity (37%), emphasizing that both serological and clinical approaches face significant limitations in accurately identifying melioidosis cases.

When compared with other diagnostic approaches in Table 1, the limitations of serological methods become more evident. Antigen-based rapid diagnostic tests (RDTs) can achieve 99% sensitivity in blood culture samples, but performance declines markedly in direct clinical specimens, with 47.1% in pus and 33.3% in sputum, indicating strong dependence on sample type [13,26,27,28]. In contrast, molecular methods such as real-time PCR demonstrate higher and more consistent performance, with sensitivity of 67.7–89.6% and specificity of 96–100% [18].

Beyond these quantitative limitations, previous studies have emphasized that serological assays are often neither sufficiently sensitive nor specific and are frequently based on unstandardized “in-house” protocols, leading to inconsistent diagnostic performance across settings [43]. Consequently, serological methods are generally regarded as adjunct tools rather than primary diagnostic approaches and are not recommended as standalone tests for routine diagnosis, particularly in endemic regions.

Taken together, although serological methods offer advantages in speed and accessibility, their diagnostic reliability is fundamentally constrained by delayed antibody response, cross-reactivity, epidemiological confounding, and interpretative limitations. Therefore, serological assays should be used cautiously and only as complementary tools alongside more specific diagnostic methods.

### 3.3. Rapid Diagnostic Tests (RDTs)

Rapid diagnostic tests (RDTs), particularly those based on lateral flow immunoassays (LFIs), have emerged as practical tools to overcome the time limitations of culture-based methods by enabling point-of-care detection of *B. pseudomallei*. These assays provide rapid results (typically within 15–30 min) and are especially valuable in resource-limited or field settings [13,26,27,28].

One of the most widely used RDTs is the Active Melioidosis Detect (AMD) test, which employs monoclonal antibodies targeting the capsular polysaccharide (CPS) antigen of *B. pseudomallei*. As summarized in Table 1, AMD demonstrates very high sensitivity in blood culture samples (~99%) with specificity approaching 100%, indicating excellent diagnostic performance under optimal conditions [13,26,27,28].

However, a critical limitation of RDTs is their strong dependence on sample type. The sensitivity of AMD is markedly lower in direct clinical specimens, with reported values of only 47.1% in pus and 33.3% in sputum, while maintaining high specificity (100%) [13,26,27,28]. This sharp decline highlights that RDT performance is not uniform and is significantly influenced by bacterial load and sample matrix complexity. Consequently, RDTs may yield false-negative results in localized infections or early-stage disease, particularly when bacteremia is absent.

These limitations are further supported by real-world clinical data. In a cohort of 232 culture-confirmed melioidosis patients, the overall sensitivity of AMD was only 56% when considering positivity from any clinical sample, indicating moderate diagnostic performance outside controlled conditions [44]. Sensitivity varied substantially by specimen type, with lower detection rates in serum (27%) compared to urine (63%), sputum (48%), and pus/tissue samples (77%) [44]. Moreover, sensitivity increased with disease severity, reaching 39% in bacteremic cases and up to 68% in septic shock, further confirming that antigen detection is closely associated with bacterial burden [44].

In addition, the detection of CPS antigen in urine, even in the absence of culture positivity, suggests that antigenuria may reflect systemic infection rather than localized urinary tract involvement, complicating clinical interpretation [44]. Another important limitation is that RDTs may detect residual antigen after antibiotic treatment, yielding positive results despite negative culture findings, thereby limiting their ability to reflect active bacterial viability.

In addition to CPS-based antigen detection, alternative RDT strategies targeting host antibody responses have also been developed. A rapid immunochromatographic test (ICT) based on the hemolysin co-regulated protein 1 (Hcp1) demonstrated 88.3% sensitivity and specificity ranging from 86.1% to 100% across different populations, indicating robust diagnostic performance [45]. Notably, this assay outperformed conventional serological methods such as the indirect hemagglutination assay (IHA) and ELISA, which showed lower sensitivity (69.5% and 83%, respectively) [45]. However, as an antibody-based diagnostic approach, its performance remains dependent on host immune response and may be limited in early infection.

In comparison with other diagnostic methods presented in Table 1, RDTs occupy an intermediate position. Their speed and simplicity offer clear advantages over culture and molecular assays; however, their sensitivity in direct clinical samples remains inferior to molecular methods such as real-time PCR, which demonstrate sensitivity of 67.7–89.6% and specificity of 96–100% across multiple sample types [18]. Similarly, monoclonal antibody-based latex agglutination assays provide rapid results with a sensitivity of 94–96.75%, but their use is largely restricted to blood culture fluid and requires prior bacterial growth [21,22,23].

Recent developments, including amplification-integrated lateral flow biosensors and improved antigen-targeting strategies, show potential to enhance RDT performance. However, these approaches remain dependent on assay design and require further validation across diverse clinical settings [27,28,45].

Taken together, although RDTs offer significant advantages in speed, ease of use, and field applicability, their diagnostic reliability is strongly influenced by sample type and bacterial load. Therefore, RDTs should not be used as standalone diagnostic tools, particularly for non-blood specimens, but rather as complementary methods alongside culture or molecular diagnostics to ensure accurate detection of melioidosis.

### 3.4. Molecular Method

Molecular methods, particularly polymerase chain reaction (PCR), are widely used to detect *B. pseudomallei* DNA in clinical samples, including blood, pus, sputum, and urine. These approaches enable rapid and highly specific detection by directly targeting bacterial genetic material. As summarized in Table 1, real-time PCR demonstrates sensitivity ranging from 67.7% to 89.6% and specificity from 96% to 100%, depending on the sample type and target gene [18,46,47,48].

Despite this high analytical performance, several studies have highlighted critical limitations in the molecular detection of *B. pseudomallei*. The species exhibits substantial genetic diversity and high recombination rates, complicating the identification of universally reliable molecular targets and potentially leading to false-positive or false-negative results [46]. This indicates that PCR performance, although generally robust, is not inherently uniform across all strains and settings.

In addition to genetic variability, PCR performance is strongly influenced by sample type. Sensitivity is often reduced in blood samples due to low bacterial load, whereas higher detection rates are typically observed in pus or abscess fluid, where bacterial concentrations are greater [18]. This pattern is consistent with other diagnostic approaches, such as RDTs, where detection efficiency is also highly dependent on bacterial burden and sample matrix complexity.

Compared with conventional diagnostic methods, PCR offers substantial advantages in turnaround time, providing results within hours rather than the days required for culture [8]. In contrast to serological assays, PCR enables direct detection of bacterial DNA and avoids cross-reactivity associated with antibody-based methods [13,14]. Furthermore, PCR demonstrates more consistent performance than RDTs, whose sensitivity varies significantly depending on sample type and bacterial burden [13,26,27,28].

However, the implementation of PCR-based diagnostics remains constrained by practical limitations, including the need for specialized laboratory infrastructure, trained personnel, and higher operational costs, which restrict their accessibility in resource-limited settings [8]. In such environments, melioidosis is frequently underdiagnosed, with reported cases likely representing only a fraction of the true disease burden due to limited diagnostic capacity [49].

PCR-based detection relies on specific genetic targets associated with *B. pseudomallei* virulence and physiology. Among these, the type III secretion system gene cluster (TTS1) and capsular polysaccharide (CPS) genes are the most widely used due to their high specificity [17,31,32,50]. Additional targets, including BPSS1187 and bimA, have been explored to enhance species-specific detection, particularly in multiplex PCR approaches, as summarized in Table 2 [18,51,52,53]. Importantly, multiplex PCR strategies that integrate multiple gene targets have been shown to improve diagnostic accuracy and enable better differentiation from closely related species [54].

Other markers, such as bpeA and fliC, may provide supplementary diagnostic information related to antibiotic resistance and bacterial motility [55,56]. Furthermore, genes such as lipA and ompA have been investigated for use in multiplex PCR strategies to improve detection performance [50,57]. The 16S rRNA gene, although widely used for universal bacterial detection due to its high sensitivity, has limited specificity for *B. pseudomallei* and therefore requires confirmatory testing [58]. In addition, hcp1, which is associated with virulence and intracellular pathogenicity, has demonstrated high sensitivity as a molecular target in PCR-based assays [18,59,60,61].

Nevertheless, no single molecular marker has demonstrated perfect diagnostic accuracy across all strain collections. Comparative studies have shown that even well-established assays, including TTS1-based detection, may occasionally misidentify when tested against genetically diverse isolates [46]. Therefore, reliance on a single genetic target may be insufficient for definitive diagnosis.

Taken together, PCR-based molecular methods provide rapid and highly specific detection of *B. pseudomallei*, making them valuable tools for early diagnosis. However, their diagnostic reliability is influenced by genetic variability, sample type, and resource availability. Consequently, the use of multiple genetic targets or multiplex approaches is strongly recommended to improve diagnostic accuracy and reduce the risk of misclassification [46,54].

**Table 2:** Molecular target gene for *Burkholderia pseudomallei*.

Target Gene	Function	Strength	Main Application	References
*TTS1*	Type III secretion system, virulence	Highly specific	PCR, qPCR, multiplex PCR	[17,31]
CPS	Capsular polysaccharide, virulence factor	High specificity	PCR, LAMP, CRISPR	[32,50]
*bpss1187*	A gene involved in the virulence of *B. pseudomallei*.	A new gene that can provide more specific detection of pathogenic strains.	Multiplex PCR, Real-time PCR for more specific detection of pathogenic strains	[18]
*bimA*	There is an association with bacterial motility and intracellular spread.	Improving the detection of pathogenic strains, since it plays a role in motility and intracellular spread.	Real-time PCR, Multiplex PCR to detect pathogenic strains and monitor infections	[51,52,53]
*bpeA*	Involved in antibiotic resistance mechanisms and virulence.	Enables detection of antibiotic-resistant strains of *B. pseudomallei*.	Real-time PCR and Multiplex PCR to detect resistant strains.	[55]
*fliC*	Flagellar gene, contributes to motility and infection.	Identifies bacteria by their motility—useful in distinguishing them from other species.	Multiplex PCR for differentiation between *B. pseudomallei* and other non-pathogenic species.	[56]
*lipA*	Lipase, lipid metabolism	Systemic infection detection	Real-time PCR	[50]
*16S rRNA*	Ribosomal RNA gene, general bacterial identification	High sensitivity, widely used	Universal bacterial detection, PCR	[58]
*hcp1*	Role in cell fusion and macrophage cytotoxicity	High sensitivity	PCR	[18,59,60,61]
*ompA*	Outer membrane protein, cell wall structure	Good combination in multiplex PCR	PCR for local and systemic infections	[57]

### 3.5. Matrix-Assisted Laser Desorption Ionization–Time of Flight Mass Spectrometry (MALDI-TOF MS)

Matrix-Assisted Laser Desorption Ionization–Time of Flight Mass Spectrometry (MALDI-TOF MS) has emerged as a rapid and reliable tool for the identification of *B. pseudomallei* in clinical microbiology laboratories. This technique is based on the ionization of bacterial proteins using a laser-absorbing matrix, followed by time-of-flight analysis of the resulting ions by mass-to-charge (m/z) ratio, generating a characteristic spectral fingerprint that can be matched against reference databases for species identification [62].

MALDI-TOF MS enables rapid identification within minutes of bacterial colony availability, significantly reducing turnaround time compared to conventional biochemical methods. Previous studies have demonstrated its potential to accurately identify *B. pseudomallei* in clinical settings [63]. Large-scale evaluations have demonstrated excellent diagnostic performance, with studies reporting up to 100% sensitivity and specificity for *B. pseudomallei* identification when appropriate reference spectra are included in the database [64].

However, the diagnostic accuracy of MALDI-TOF MS is highly dependent on the completeness and representativeness of the reference database. Inadequate databases may lead to frequent misidentification, particularly confusion with closely related species such as *B. thailandensis*. For example, studies have shown that isolates may be incorrectly identified when the database lacks sufficient reference spectra, whereas expanding the database with additional strains significantly improves identification accuracy [62]. Furthermore, the inclusion of geographically diverse isolates is essential to capture intraspecies variability and enhance diagnostic reliability [62].

Despite its advantages, MALDI-TOF MS still has several limitations. The method requires prior bacterial culture and a sufficient bacterial load for accurate identification, typically requiring at least 10^7^ CFU/mL. In blood culture samples, reliable identification is generally achieved only after approximately 24 h of incubation [64]. These constraints limit its utility for early-stage detection and highlight the need for complementary rapid diagnostic approaches.

### 3.6. Loop-Mediated Isothermal Amplification (LAMP)

LAMP is a well-established technique for DNA amplification, with excellent potential for detecting *B. pseudomallei*, especially in resource-limited settings. This technique proceeds at an isothermal temperature of 60–65 °C, using four to six primers targeting particular regions of bacterial DNA, and hence, gives rise to rapid and efficient amplification without the need for an expensive thermal cycler. One of the key advantages of LAMP is its ability to provide results in less than an hour, making it highly relevant for managing acute infections such as melioidosis [65,66]. The LAMP assay targeting specific *B. pseudomallei* genes has demonstrated >95% sensitivity and high specificity, detecting as few as 38 genome copies per reaction [31]. Its rapidity and minimal equipment requirements make it well-suited for early detection of melioidosis in endemic, resource-limited areas. Future studies will aim to improve the efficiency of LAMP by addressing primer stability and contamination issues and generalizing the technique across all clinical settings. However, LAMP also has notable limitations. The method’s high sensitivity increases the risk of cross-sample contamination, potentially leading to false positives. Additionally, LAMP primer design is complex and requires more extensive optimization compared to conventional PCR. Detection based on colorimetric or fluorescent changes can be subjective and often necessitates confirmatory testing with more definitive diagnostic methods [31]. Despite the availability of various diagnostic approaches, limitations related to sensitivity, turnaround time, cross-reactivity, and laboratory infrastructure continue to hinder the early detection of melioidosis. These challenges highlight the need for more specific and reliable biomarkers, which has driven increasing interest in antigen-based targets and short peptide epitopes derived from key *B. pseudomallei* virulence factors.

## 4. Diagnostic Biomarkers of *Burkholderia pseudomallei*

### 4.1. Diagnostic Biomarker Candidates in B. pseudomallei

In addition to the well-established diagnostic targets CPS and TTS1, several other antigenic proteins of *B. pseudomallei*, including BPSS1187, BimA, Hcp1, LipA, and OmpA, have been investigated as potential biomarkers, particularly for short peptide–based diagnostics. However, their clinical validation remains limited, as summarized in Table 3 [18,50,51,52,53,57]. Among these candidates, secreted proteins such as Hcp1 (a component of the type VI secretion system), LipA, and other effector proteins have attracted particular interest because they can be detected in biological fluids. Unlike intracellular targets, these proteins may be released into blood, urine, or sputum without requiring bacterial lysis, making them attractive for non-invasive or early diagnostic approaches [18,67]. For example, Hcp1 has been detected in the serum of patients with melioidosis and used in immunoassay-based diagnostics, demonstrating its relevance as a clinically accessible biomarker [18,58,59,60].

Despite these advantages, the diagnostic performance of secreted protein biomarkers is constrained by several critical factors. First, their concentration in clinical samples is often low, particularly during early infection, which may limit detection sensitivity [45]. Second, these proteins are susceptible to degradation by host proteases, reducing their stability and detectability. Third, their expression and secretion are influenced by environmental conditions, including pH, temperature, and bacterial growth phase, leading to variability in diagnostic performance [31]. Collectively, these factors contribute to inconsistent detection and limit their reliability as standalone diagnostic targets.

Specificity also remains a major challenge. For instance, LipA and other enzymatic proteins may be conserved across related *Burkholderia* species or environmental bacteria, increasing the risk of cross-reactivity and false-positive results. Similarly, while OmpA is immunogenic and structurally important, its diagnostic performance has not been clinically validated; existing evidence is largely limited to experimental or immunogenicity studies [57].

In comparison, CPS and TTS1 remain the most reliable diagnostic targets due to their higher specificity and more consistent performance across clinical studies. CPS, as a surface-associated polysaccharide, is widely used in both molecular and antigen-based assays and is strongly associated with active infection, although its sensitivity may vary depending on sample type and bacterial load [68,69]. Likewise, TTS1-based detection demonstrates high specificity and has been extensively validated in PCR-based diagnostics, although its performance may still be influenced by environmental and sample-related factors [18].

Notably, emerging targets such as BPSS1187 have shown promising specificity and relatively high sensitivity in molecular assays; however, their diagnostic utility remains dependent on bacterial load and stage of infection, and further validation is required [18]. Similarly, BimA, despite its role in virulence and intracellular motility, has not yet been validated as a diagnostic biomarker and lacks reported data on clinical sensitivity and specificity [51,52,53].

Taken together, although multiple antigenic candidates have been explored, most remain at an early stage of validation and exhibit limitations in sensitivity, specificity, and stability. Consequently, CPS and TTS1 remain the most robust and clinically reliable diagnostic targets. Future biomarker development should focus on improving detection sensitivity, minimizing cross-reactivity, and integrating multiple targets or peptide-based epitopes to enhance diagnostic accuracy, particularly in resource-limited settings.

### 4.2. Key Biomarkers: CPS and TTS1

CPS and TTS1 are widely recognized as the most reliable diagnostic biomarkers for *B. pseudomallei*, primarily due to their high specificity and relative genetic conservation across diverse strains [17,31]. Genomic analyses indicate that both loci are broadly conserved, supporting consistent detection across clinical isolates. However, given the genomic plasticity and recombination capacity of *B. pseudomallei*, the assumption of long-term invariability remains uncertain, and continuous surveillance of these targets is necessary to ensure sustained diagnostic reliability [31,32].

CPS has been extensively utilized in antigen-based and molecular assays, including real-time PCR, lateral flow immunoassays (LFI), and ELISA, with reported sensitivity exceeding 95% and specificity approaching 100% under optimized conditions [68,70]. Despite this strong performance, CPS-based detection is not without limitations. Structural similarity between CPS of *B. pseudomallei* and those of closely related species, such as *B. thailandensis*, may compromise specificity, particularly in endemic regions where coexistence increases the likelihood of cross-reactivity [68,70]. In addition, CPS is subject to biological dynamics such as rapid clearance from circulation, which may restrict the diagnostic window and contribute to false-negative results if sampling is not appropriately timed [69]. To overcome these limitations, CPS-derived peptide fragments have been proposed as alternative diagnostic targets, offering improved stability and potentially enhanced detection performance, particularly in resource-limited settings [71].

In contrast, TTS1 represents a highly specific molecular target with no reported cross-reactivity with non-pathogenic *Burkholderia* species, making it particularly suitable for nucleic acid–based diagnostics. As a component of the type III secretion system (T3SS), TTS1 plays a central role in virulence and is among the most consistently conserved genetic regions in *B. pseudomallei*, supporting its use in real-time PCR and LAMP assays [31]. Short peptide–based detection strategies derived from TTS1 have also demonstrated high diagnostic performance, with sensitivity exceeding 95% and specificity approaching 100% in ELISA and LFI platforms [31,55]. These findings highlight the dual applicability of TTS1 in both molecular and peptide-based diagnostic approaches.

Beyond CPS and TTS1, several additional targets have been explored to expand the diagnostic landscape. BPSS1187 has shown promising performance in molecular assays, with a sensitivity of 89.6% and a specificity of 96% in multiplex PCR and LAMP applications [18]. However, its translation into peptide-based diagnostics remains limited and requires further validation. Similarly, BimA, a virulence-associated protein involved in intracellular motility, has been investigated as a molecular target, but its diagnostic performance has not been systematically validated, particularly in peptide-based formats [51,52,53].

A critical distinction between CPS and TTS1 lies in their diagnostic modality and biological behavior. CPS is advantageous for antigen-based detection in non-invasive samples such as urine; however, its rapid renal clearance and variable abundance may limit its reliability in early or low-burden infections [69]. In contrast, TTS1 provides a stable, highly specific genetic target that is less affected by biological variability, making it more suitable for molecular platforms that prioritize sensitivity and reproducibility. This distinction underscores the complementary nature of these biomarkers rather than their interchangeability.

Taken together, while emerging biomarkers such as Hcp1, BPSS1187, and BimA contribute to expanding diagnostic possibilities, CPS and TTS1 remain the most robust and clinically validated targets. Their combined use—integrating antigen-based and molecular detection—offers a more reliable diagnostic strategy by balancing sensitivity, specificity, and practical applicability. Future advances should focus on integrating these targets into multiplex or peptide-based platforms, improving stability and detection limits, and validating performance across diverse clinical and epidemiological settings.

## 5. Short Peptide Biomarkers for Melioidosis Diagnostics

Despite increasing interest in peptide-based diagnostics, experimentally validated short peptide biomarkers for *B. pseudomallei* remain scarce, highlighting a gap between antigen-based biomarker discovery (Section 4) and peptide-level diagnostic development. Most current evidence still relies on well-characterized antigenic targets, particularly capsular polysaccharide (CPS), type III secretion system 1 (TTS1), and several virulence-associated proteins such as Hcp1 and BPSS1187 [17,18,31,32,50,68,69]. While these antigens provide a strong foundation for epitope-based design, direct validation of peptide-derived diagnostic biomarkers remains limited.

Building on the limitations of antigen-based diagnostics discussed in Section 3 and Section 4, peptide-based diagnostics are needed to address the inherent constraints of existing methods. As discussed in Section 3 and Section 4, current diagnostic approaches are constrained by sample-type dependency, reduced sensitivity in non-blood specimens, cross-reactivity with closely related *Burkholderia* species, and the requirement for advanced laboratory infrastructure [13,18,26,27,28,45,68,70]. Short peptides offer a potential solution to these challenges by providing highly specific, stable, and synthetically accessible recognition elements. Unlike full-length proteins or polysaccharides, peptides can be designed to target species-specific epitopes, thereby reducing cross-reactivity while maintaining diagnostic precision [72,73,74].

From a biochemical perspective, short peptides are defined as epitope fragments that can interact with antibodies or immune receptors with high specificity. Their small size enables improved stability, reduced susceptibility to degradation, and reproducible large-scale synthesis. In addition, peptide-based biomarkers are highly adaptable across diagnostic platforms, including ELISA, lateral flow immunoassays (LFI), and nanoparticle-based biosensors, making them particularly suitable for point-of-care applications in resource-limited settings [72,73,74].

However, the application of peptide-based diagnostics in melioidosis remains at an early developmental stage. Current studies are largely focused on in silico epitope prediction, antigen prioritization, and preliminary immunoreactivity screening, with limited translation into clinically validated diagnostic assays. This gap highlights several unresolved challenges, including the identification of immunodominant and species-specific epitopes, optimization of peptide stability under physiological conditions, and validation across diverse patient populations [72,73,74].

Among available targets, CPS- and TTS1-derived epitopes remain the most promising due to their established diagnostic relevance and relative specificity. CPS-derived peptides may offer improved stability and reduced dependence on antigen dynamics, such as rapid clearance, whereas TTS1-derived peptides benefit from high genetic specificity and minimal cross-reactivity. Additional candidates, including Hcp1, BPSS1187, and BimA, may provide complementary epitopes; however, their diagnostic performance remains insufficiently validated and requires further investigation [17,18,31,32,51,52,53,56,68,69].

Taken together, peptide-based diagnostics for melioidosis represent a promising but still emerging field. While strong antigenic foundations have been established, the transition toward clinically applicable peptide biomarkers requires systematic validation, integration of multi-epitope strategies, and optimization for field-deployable platforms. Future research should prioritize identifying highly specific peptide epitopes and incorporating them into robust, cost-effective diagnostic systems suitable for endemic and resource-constrained settings.

### 5.1. Advantages of Short Peptides in Diagnostic Applications

Targeting immunogenic epitopes using short peptides has been proposed as an effective strategy to enhance the diagnostic specificity and stability of *B. pseudomallei* detection [50]. Compared with full-length proteins or complex antigens, peptide-based biomarkers offer several key advantages, including improved production efficiency, higher stability, and enhanced specificity.

One of the primary advantages of short peptides lies in their synthetic accessibility. Peptides can be readily produced using solid-phase peptide synthesis, enabling rapid, scalable, and standardized production. In addition, their chemical structure allows targeted modifications—such as cyclization or terminal protection—to improve stability and binding specificity. This contrasts with whole antigens, including lipopolysaccharides (LPS), which are more prone to structural variability and cross-reactivity with closely related species [75,76].

Short peptides also exhibit superior stability compared with full-length proteins. Unlike proteins that require complex folding and are susceptible to denaturation, peptides generally maintain structural integrity under harsh environmental conditions, including elevated temperatures and suboptimal storage. This characteristic makes them particularly suitable for diagnostic applications in resource-limited and tropical settings, where cold-chain infrastructure may be limited [77].

Importantly, the small size and sequence specificity of peptides enable precise targeting of unique epitopes derived from key *B. pseudomallei* antigens such as CPS and TTS1. This targeted recognition can enhance diagnostic sensitivity while minimizing cross-reactivity with closely related species, including *B. thailandensis* [49]. In this context, peptide-based approaches provide a rational strategy to overcome one of the major limitations of antigen-based diagnostics, namely, nonspecific binding and false-positive results.

Despite these advantages, peptide-based diagnostics also present several practical challenges. Short peptides may be susceptible to enzymatic degradation in biological samples, exhibit limited immunogenicity in certain contexts, and show variable performance across different sample matrices. Furthermore, maintaining peptide stability during storage and transport—particularly in endemic regions with high temperatures and humidity—remains a critical consideration for field implementation [72,73,74].

A key challenge in melioidosis diagnostics is identifying epitopes that reliably distinguish *B. pseudomallei* from closely related species, particularly *B. thailandensis* and other environmental *Burkholderia* spp. [32,38,68,70]. This highlights the necessity for rigorous epitope selection strategies to ensure high diagnostic specificity.

From a biomarker development perspective, epitope conservation must be carefully evaluated to avoid selecting peptide regions shared across related species. Bioinformatic approaches, including sequence alignment and comparative genomics, play a crucial role in identifying species-specific peptide candidates. However, computational prediction alone is insufficient; experimental validation using panels of closely related *Burkholderia* species is essential to confirm specificity and minimize cross-reactivity in clinical applications [32,38,68,70].

### 5.2. Recent Applications of Short Peptides in Diagnostic Platforms

To further illustrate the practical implications of peptide-based diagnostics, recent applications of short peptides across various infectious diseases have been explored [78]. Their compatibility with multiple diagnostic platforms—including ELISA, lateral flow immunoassays (LFI), and nanoparticle-based biosensors—enables flexible implementation in both laboratory-based and point-of-care settings [69,79]. Importantly, these applications consistently demonstrate that peptide-based recognition can improve assay specificity and reduce diagnostic time compared with conventional antigen-based approaches.

A key principle underlying peptide-based diagnostics is the ability to target specific immunogenic epitopes rather than whole antigens. For example, in *Mycobacterium tuberculosis*, peptides derived from the ESAT-6 protein have demonstrated improved specificity by focusing on immunodominant regions associated with infection [80,81]. This epitope-level targeting reduces cross-reactivity—a limitation that is also highly relevant in melioidosis diagnostics, particularly in distinguishing *B. pseudomallei* from closely related species.

Recent studies further demonstrate that peptide-based diagnostic platforms can achieve rapid, sensitive detection across diverse technological modalities. For instance, Magainin I has been used to detect *Escherichia coli* O157, achieving detection limits of approximately 10^3^ CFU/mL within 90 min with electrochemical biosensors, and even lower limits in food matrices with colorimetric assays [82,83]. Similarly, peptide-functionalized biosensors targeting *Pseudomonas aeruginosa* and *Listeria monocytogenes* have demonstrated detection limits ranging from 10^5^ CFU/mL to 200 CFU/mL, depending on the sensing platform and peptide design [69,84]. These examples highlight the adaptability of peptide-based diagnostics across diverse platforms and sample types.

However, it is important to emphasize that these studies are based on pathogens other than *B. pseudomallei* and therefore primarily demonstrate the feasibility of peptide-based diagnostic strategies rather than their established application in melioidosis. In the case of *B. pseudomallei*, direct experimental validation of short peptide biomarkers remains limited, and, to date, no short peptide biomarker has been fully validated for routine clinical diagnostic use. This discrepancy underscores a critical translational gap: while peptide-based diagnostics have been successfully applied in other bacterial systems, their application in melioidosis remains largely conceptual and requires further investigation. Future studies should therefore focus on translating well-characterized antigenic targets, such as CPS and TTS1, into validated peptide-based diagnostic platforms with demonstrated clinical performance.

## 6. Epitope Mapping of Carbohydrate and Protein Antigens

Epitope mapping plays a critical role in the development of peptide-based diagnostic assays by enabling the identification of antigenic regions for the specific and sensitive detection of *B. pseudomallei* [30,69,85]. In this context, epitope selection is not only important for immunological recognition but also for ensuring diagnostic accuracy and minimizing cross-reactivity [69,86]. Identification of short diagnostic peptides requires accurate determination of antigenic epitopes. Therefore, understanding epitope mapping strategies is essential for selecting peptide sequences with high diagnostic specificity and sensitivity. The intrinsic nature of antigens determines the strategies used for epitope mapping. For carbohydrate-based antigens such as capsular polysaccharides (CPS), immunogenic epitopes are generally conformational and depend strongly on three-dimensional structural arrangements. Unlike proteins, which contain predictable linear amino acid sequences and well-defined tertiary structures, carbohydrate antigens often exhibit complex, highly branched conformational architectures. This structural complexity makes epitope prediction for carbohydrate antigens more challenging and often more dependent on experimental approaches than on in silico analysis. In pathogens such as *B. pseudomallei*, limited structural information further complicates computational prediction of carbohydrate epitopes. In addition, carbohydrate epitopes are highly conformation-dependent, meaning that antibody recognition is strongly influenced by the three-dimensional organization of polysaccharide structures.

Monoclonal antibodies are commonly used to map specific epitopes on polysaccharide antigens through experimental techniques such as ELISA, Western blotting, or immunoprecipitation assays [69]. Unlike protein antigens, epitope mapping of CPS typically focuses on identifying immunogenic glycosidic motifs within polysaccharide fragments. These motifs can be analyzed using tools such as the GlycoEpitope database (available online), often after enzymatic or chemical fragmentation, including periodate oxidation. The resulting fragments can then be evaluated using glycan array technology to validate antigen–antibody interactions [85]. This approach has been applied in studies of carbohydrate antigens, particularly in glycoprotein-based vaccine development [71]. Structural validation using nuclear magnetic resonance (NMR) spectroscopy or X-ray crystallography is often required to confirm the three-dimensional structures of carbohydrate epitopes that interact with immune receptors [69,71]. Despite these advances, carbohydrate epitope mapping remains challenging due to the structural diversity and conformational complexity of polysaccharides, which often requires a multidisciplinary analytical approach [49].

Glycan array technology further enables the synthesis and screening of defined carbohydrate structures, allowing precise identification of antibody-recognized epitopes and facilitating large-scale analysis of glycan–antibody interactions [85]. This technology has the potential to significantly improve the sensitivity and specificity of diagnostic assays [49]. However, the application of glycan array technology remains limited to laboratories with advanced analytical facilities. In addition, results from glycan array screening usually require further validation using immunological techniques, such as ELISA, to confirm their clinical relevance.

In contrast, epitopes derived from protein antigens, including those associated with the TTS1 secretion system, may consist of either linear amino acid sequences or conformational epitopes. Consequently, in silico approaches play an important role in predicting protein epitopes using bioinformatics tools such as the Immune Epitope Database (IEDB) Analysis Resource, BepiPred, and NetMHCII. The IEDB Analysis Resource is widely used to identify epitopes on protein antigens and to support immune-based diagnostics and vaccine development by analyzing interactions among epitopes, antibodies, and major histocompatibility complex (MHC) molecules. The platform can also integrate three-dimensional structural data from the Protein Data Bank (PDB) to improve the accuracy of epitope prediction [85].

For B-cell epitope prediction, algorithms such as BepiPred 2.0 are commonly used to identify potential linear epitopes from amino acid sequences and discontinuous epitopes located on protein surfaces [85]. In addition, tools such as NetMHCpan are used to predict T-cell epitopes that bind to class I and class II MHC molecules, thereby contributing to cellular immune responses [87]. The integration of structural information enables the identification of conformational epitopes on protein antigens associated with TTS1. One of the strengths of the IEDB platform is its ability to prioritize epitopes based on parameters such as immunogenicity, binding affinity, and HLA allele distribution, thereby improving the likelihood of identifying clinically relevant targets.

Experimental validation remains essential to confirm computational predictions. Techniques such as peptide scanning are frequently used, in which short peptide fragments derived from target proteins are synthesized and evaluated for antibody binding using peptide ELISA or microarray assays [31]. These approaches are particularly relevant because TTS1-associated proteins are linked to bacterial pathogenicity and may serve as important molecular targets for diagnostic detection [88]. In addition to linear epitope prediction, conformational epitopes can be identified using structural bioinformatics tools such as DiscoTope 2.0, based on three-dimensional protein models derived from homology modeling or crystallographic data [89]. Structural validation of conformational epitopes is typically performed using techniques such as X-ray crystallography, cryo-electron microscopy (Cryo-EM), or NMR spectroscopy to identify specific regions of proteins that interact with antibodies [90].

Previous studies have shown that TTS1-related proteins represent highly sensitive diagnostic targets due to their strong genetic conservation across diverse *B. pseudomallei* strains. Overall, the differences in epitope determination between carbohydrate and protein antigens reflect the distinct physicochemical characteristics of each antigen type, as summarized in Table 4. In *B. pseudomallei*, integrating computational prediction and experimental validation strategies for CPS and TTS1-associated antigens may facilitate more accurate epitope identification and support the development of short peptide–based diagnostic platforms with improved sensitivity and specificity [69,88]. From a diagnostic perspective, the selection of well-defined, specific epitopes is essential for developing reliable platforms such as ELISA, lateral flow assays, and biosensor-based detection systems [30,66,85]. Therefore, epitope mapping should be viewed as a translational step that directly bridges antigen discovery and practical diagnostic implementation [69,88].

## 7. Conclusions

CPS and TTS1 remain the most well-established diagnostic targets for *B. pseudomallei*, with demonstrated performance in antigen-based and molecular diagnostic platforms. While these targets provide a strong foundation for diagnostic development, their translation into short peptide–based biomarkers remains limited.

This review highlights the progression from conventional diagnostic approaches—often constrained by limitations in speed, sensitivity, and field applicability—toward peptide-based strategies as a potential future direction. Short peptides derived from CPS and TTS1 offer theoretical advantages in terms of specificity, stability, and adaptability across diagnostic platforms. However, it is important to emphasize that peptide-based diagnostics for *B. pseudomallei* remain in the early stages of development, and, to date, no short peptide biomarker has been fully validated for routine clinical use.

Therefore, peptide-based diagnostics should currently be viewed as an emerging approach rather than an established solution. Future research should focus on rigorous epitope selection, experimental validation, and the development of robust, field-deployable platforms to translate these candidates into clinically applicable diagnostic tools for melioidosis.

## Figures and Tables

**Table 1:** Comparison of current diagnostic methods for Burkholderia pseudomallei detection.

Diagnostic Method	Mechanism	Sample Type	Study Population	Strengths	Limitations	Sensitivity	Specificity	Ref.
Culture	Isolation of *B. pseudomallei* from clinical specimens	throat swab	1011 melioidosis patients; 3524 controls	Gold standard; high specificity	Low sensitivity; depends on sample type	36.1%	100%	[17]
Culture (general)	Isolation of bacteria from clinical samples	Blood, sputum, pus	Patients with suspected melioidosis	Definitive diagnosis	Slow; requires lab facilities	Variable	High (~100%)	[8]
Serology (IHA/ELISA)	Detection of antibodies	Serum	Endemic population	Simple	Cross-reactivity; poor specificity	Variable	Variable	[8]
real-time PCR	Detection of specific DNA targets (e.g., TTS1, BPSS1187)	Plasma, urine, sputum, pus	Patients with suspected and culture-confirmed melioidosis	High specificity, rapid detection, and detects early infection	Reduced sensitivity in blood samples requires laboratory infrastructure	67.7–89.6% (depending on sample type and target gene)	96–100%	[18]
Monoclonal antibody-based latex agglutination	Detection of *B. pseudomallei* antigens using monoclonal antibody-coated latex particles	Blood culture fluid	Patients with community-acquired septicemia (multi-center study)	Rapid (<5 min), simple, high accuracy in blood culture fluid	Requires specific monoclonal antibodies, limited applicability to direct clinical samples (requires prior culture)	94–96.75%	83–99.7%	[21,22,23]
Automated identification systems (API 20NE, API 20E, VITEK)	Biochemical profiling using manual or automated systems	Clinical isolates	*B. pseudomallei* isolates from clinical cases (*n* = 103)	Widely used in diagnostic laboratories; automated workflow	Significant variability in accuracy; high risk of misidentification due to database limitations	37–99%	92% or variable	[24,25]
Serological Methods (ELISA/IHA)	Detection of antibodies/antigens	serum	Individuals in endemic and non-endemic settings, patients with suspected melioidosis	Fast, easy to use, suitable for latent detection	Cross-reactivity, low sensitivity	~50–60%	~80–90%	[13,14]
Rapid Diagnostic Tests (RDTs)	Detection of *B. pseudomallei* capsular polysaccharide (CPS) antigen using monoclonal antibody-based lateral flow immunoassay	Blood culture, pus, sputum, urine,	Patients with suspected and culture-confirmed melioidosis (prospective clinical evaluation)	Rapid (<15–30 min), simple, suitable for field settings, and excellent performance in blood culture samples	Markedly reduced sensitivity in direct clinical specimens (e.g., sputum, sterile fluids); performance highly dependent on sample type; potential cross-reactivity with closely related *Burkholderia* species; influenced by low bacterial load and sample matrix complexity	99% (Blood culture); 47.1% (pus); 33.3% (sputum); 86.7% (urine)	100% (Blood culture); 100% (pus); 100% (sputum); 90.7% (urine)	[13,26,27,28]
Molecular (PCR)	Detection of DNA	Clinical samples	Clinical cases	Rapid; sensitive	Cost; lab requirement	High	High	[8,29,30]
LAMP	DNA amplification at constant temperature using strand-displacing polymerase	Blood, plasma, serum, urine, sputum	Patients with suspected and culture-confirmed melioidosis (clinical and laboratory studies)	Rapid detection, no thermal cycler required, suitable for resource-limited settings, high analytical sensitivity	Reduced sensitivity in clinical samples, particularly blood, due to low bacterial load; performance highly dependent on sample type; potential contamination leading to false positives; requires further clinical validation	44–90% (depending on study and sample type)	98–100%	[31]

**Table 3:** Potential biomarker candidates on *B. pseudomallei*.

Protein	Diagnostic Method	Sensitivity (%)	Specificity (%)	Strengths	Limitations	References
Hemolysin Co-Regulated Protein 1 (Hcp1)	ICT-based serology	88.3	86.1% (Thai healthy donors)/100% (U.S. donors)	Easily detectable inpatient serum and other bodily fluids	Low concentration in early infection, limited sensitivity	[18]
Outer Membrane Protein A (OmpA)	PCR, ELISA	Not reported	Not reported	Immunogenic outer membrane protein involved in host–pathogen interaction and structural integrity	No validated diagnostic performance reported; current evidence is limited to experimental and immunogenicity studies	[57]
Lipase A (LipA)	Enzymatic test	Not reported	Not reported	Associated with bacterial enzymatic activity and virulence	Diagnostic sensitivity and specificity not reported; clinical applicability as a biomarker remains unclear	[50]
CPS (Capsular Polysaccharide)	Real-time PCR, LAMP	>95	~100	Established diagnostic target used in molecular and antigen-based assays; associated with active infection	Sensitivity may vary depending on sample type; potential cross-reactivity with closely related *Burkholderia* species	[68,69]
TTS1 (Type III Secretion System)	Real-time PCR, Multiplex PCR	79.2% (patient-based evaluation); 67.7% (plasma)	99% (patient-based evaluation); 100% (plasma)	Highly specific for *B. pseudomallei*, genetic stability.	Affected by environmental conditions (pH, temperature)	[18]
BPSS1187	Multiplex PCR, LAMP	89.6%	96	Specific for *B. pseudomallei*, highly conserved.	Sensitivity may be influenced by bacterial load and stage of infection	[18]
BimA	Real-time PCR, Multiplex PCR	Not reported	Not reported	Virulence-associated protein involved in actin-based motility and intracellular spread	Not validated as a diagnostic biomarker; no clinical sensitivity or specificity reported	[51,52,53]

**Table 4:** Differences in the epitope determination method for carbohydrate and protein.

Aspect	Carbohydrate Antigen (CPS)	Protein Antigen (TTS1)	References
Structural characteristics	Complex polysaccharide structure; highly conformational	Linear amino acid sequences and defined three-dimensional protein structures	[69]
Types of epitopes	Primarily conformational epitopes (3D)	Linear epitopes (amino acid sequences) and conformational epitopes (3D)	[89]
Epitope prediction methods	Limited in silico prediction; mainly relies on experimental mapping	In silico prediction using tools such as IEDB, BepiPred, and NetMHCII	[85,87]
Experimental validation methods	Monoclonal antibody-based assays (ELISA, Western blot, glycan array)	Peptide scanning, ELISA, and peptide microarray	[31]
Structural characterization techniques	NMR spectroscopy, X-ray crystallography	X-ray crystallography, Cryo-EM, NMR spectroscopy	[90]
Major challenges	Structural complexity and limited computational tools for conformational prediction	Structural variability and the need for extensive experimental validation	[89]

## Data Availability

Not applicable.
